# Should Health Professionals Allow Reporters Inside Hospitals and Clinics at Times of Natural Disasters?

**DOI:** 10.1371/journal.pmed.0020177

**Published:** 2005-06-28

**Authors:** Anant Bhan

## Abstract

Journalists and health workers need to carefully consider whether it is ethical to show images of patients in medical settings, or of dead bodies in morgues.

## Teaser

Health professionals must protect their patients' dignity and privacy

**Figure:**
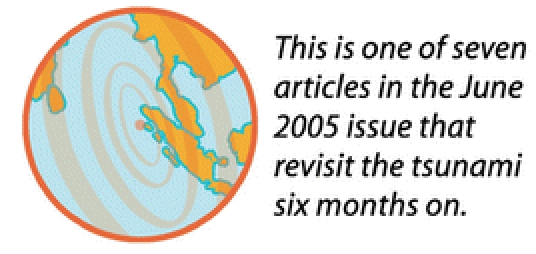


The tsunami that marked a solemn end to 2004 left behind unprecedented devastation. The world was shocked at the increasing casualty figures and the real-time images of the disaster brought by the news media. These included clips and photographs of dead bodies, grieving relatives, and suffering patients admitted to makeshift emergency wards.

The photographs did help in organizing a quick response from the rest of the world, as societal pressure led governments and relief agencies to respond with comprehensive relief measures. Graphic footage and newspaper headlines continue to dwell on this human tragedy. However, both health-care workers and journalists need to carefully consider whether it is ethical to show images of patients in obvious distress and undergoing medical attention in emergency camps, or of dead bodies in hospital morgues.

## Ethical Guidelines

There are many published guidelines that journalists can turn to for guidance on the ethics of reporting. For example, the UNESCO International Principles of Professional Ethics in Journalism details the principle of respect for privacy and human dignity as an integral part of the professional standards of a journalist [1]. The Australian Journalists Code of Ethics calls upon journalists to respect private grief and personal privacy, and reinforces the right of journalists to resist their compulsion to intrude [2]. The Code of Ethics and Professional Conduct of the Radio-Television News Directors Association, the world's largest professional organization devoted exclusively to electronic journalism, expects professional electronic journalists to treat all subjects of news coverage with respect and dignity, showing particular compassion to victims of crime and tragedy [3]. [Fig pmed-0020177-g001]

**Figure pmed-0020177-g001:**
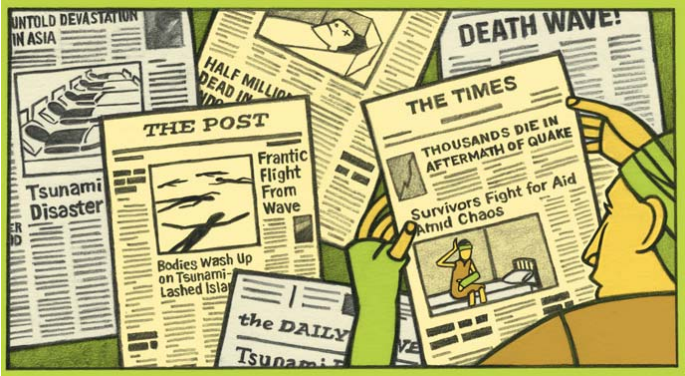
During disasters, journalists and health professionals must respect patients' privacy (Illustration: Giovanni Maki)

However, there has been little consideration to date of the ethics of health-care staff allowing access to media inside medical institutions at times of natural disasters. In a 2003 editorial in the BMJ, which discussed “man-made” disasters such as war, Singh and DePellegrin questioned the use of footage of casualties from the Iraq war without the patients' consent [4]. An extensive debate followed publication of the commentary (see http://bmj.bmjjournals.com/cgi/eletters/326/7393/774); one of the views expressed was the need to show the world the extent of killing and maiming in the war (http://bmj.bmjjournals.com/cgi/eletters/326/7393/774#31147).

## Media Coverage of the Tsunami: Benefits and Harms

In the post-tsunami scenario, the usefulness of the Internet and media was apparent. For example, a young Swedish child separated from his family was identified by his uncle on a hospital Web site and later reunited in an emotional moment with his father, who had been admitted to another hospital. The publication on government and hospital Web sites of the names of those admitted to hospitals, together with news releases, helped many identify their friends and relatives.

Furthermore, the aid response has been the largest of any disaster in history, which may have been due to the unprecedented media coverage. There has also been the advent of “disaster tourism”—the massive inflow of well-meaning, but often ill-organized, charitable organizations and aid volunteers to the tsunami-hit areas [5].

At the same time, the media coverage of wailing relatives and dead bodies lying in hospital morgues is deeply disturbing. The death of a loved one is a time for privacy and respect for the dead. As a South Asian, I am aware that in many communities the dead body is covered with a shroud that denotes purity. It is rare to photograph or film funerals. To infringe on the privacy of families when they are emotionally shattered is disrespectful to the living. Photographing and filming the deceased in various stages of undress and decomposition violates the dead and their dignitary rights, according to most cultures. In addition, the hordes of news media that descend on a hospital can hamper the efficiency of the medical staff providing emergency care, where even seconds are crucial.

## The Role of Health Professionals in Protecting Privacy

Health professionals and administrators can and should control media access to hospitals and clinics. The public's right to information should not outweigh the right of victims of natural disasters to privacy, confidentiality, and dignity. Health professionals should be aware that the filming of patients under their care may be used not only for highlighting the extent of a disaster's human toll, but also for commercial purposes, such as selling programs and newspapers, and for raising funds. For these reasons, extreme caution should be used in giving permission to use images from inside hospitals in disaster-affected areas. Ideally, the consent of the patients or surrogate decision makers should be sought first.

It is now the ethical norm to seek consent of patients when photographs of them (or even of their body parts) are used in medical conferences or publications (see the guidelines on consent from the International Committee of Medical Journal Editors, at http://www.icmje.org/#privacy). A similar approach should be taken in the event of natural or man-made disasters. If photographs of the dead or those admitted to hospitals have to be publicized for identification purposes, this should be done keeping local sensibilities in mind.

It is difficult for health-care professionals to shoulder this social responsibility during a crisis when lifesaving measures come first. Community consent and monitoring through community leaders, tribal elders, or local authorities might be an option. Such community involvement would result in media coverage that would be socially and culturally acceptable. While the usefulness of documenting and transmitting such geographically and experientially diverse experiences around the world is undeniable, the terms of access for media have to be negotiated keeping the notion of consent central.

With the increasing focus in medicine and bioethics on individual rights, the right to privacy is pivotal. Doctors and other health professionals have a duty of care to their patients, which includes protecting their dignity and privacy. Ethical obligations of health professionals to monitor recording of images in health institutions need to be higher than those of society in other venues, such as the street or the beach.

It may be valuable for medical professionals to have a specific code, perhaps written by disaster-relief organizations (such as the Red Cross) together with the World Medical Association, that outlines how to deal with the media in disaster settings. Arguably, the universal obligation of health-care professionals and administrators to respect the privacy and confidentiality of their patients should suffice, but given the nature of realities on the ground in disasters and emergencies, a specific code would be useful.

## Responsible Journalism

Responsible journalism in health-care settings at times of disaster, facilitated by guidelines that specifically address the ethical reporting of disasters and that are applicable universally across the world, will also help prevent exploitation of victims of a calamity. Such guidelines could be developed by a joint body comprised of international medical humanitarian agencies such as the Red Cross and Médecins sans Frontières (MSF), multinational agencies such as the United Nations, media representatives, and media watchdogs.

The guidelines need to be acceptable to the global media community and also need to be made binding. For example, sanctions could be imposed upon journalists (or their parent organizations) who ignore them, or perhaps only those journalists who have been accredited in “ethical reporting of disasters” should be given access to disaster sites.

The guidelines could also usefully be published together with a code for health professionals. An example of joint guidelines on ethical reporting on health issues for the media and health-care professionals are those adopted in Washington State (http://www.wsma.org/news/guide.html). These guidelines were jointly approved and prepared by media, publishers, broadcasters, and hospital and medical associations, and they could serve as a template for international guidelines on disaster reporting.

## Conclusion

In disasters, the affected are often left with almost nothing and with negligible negotiating power. They might be left with only their pride and dignity, and they must not be robbed of that. Patients or affected families might not be in a condition to respond to encroachment on their rights. While health professionals want to facilitate recognition of their unidentified patients and also facilitate more aid to affected areas, they also have an enhanced responsibility to protect their patients' dignity and rights. We should not need to be voyeurs into the grief of vulnerable victims to launch an effective and humane response to any disaster.
